# Global Distribution of the Reniform Nematode Genus *Rotylenchulus* with the Synonymy of *Rotylenchulus* *macrosoma* with *Rotylenchulus* *borealis*

**DOI:** 10.3390/plants10010007

**Published:** 2020-12-23

**Authors:** Juan E. Palomares-Rius, Ilenia Clavero-Camacho, Antonio Archidona-Yuste, Carolina Cantalapiedra-Navarrete, Guillermo León-Ropero, Sigal Braun Miyara, Gerrit Karssen, Pablo Castillo

**Affiliations:** 1 Instituto de Agricultura Sostenible (IAS), Consejo Superior de Investigaciones Científicas (CSIC), Avda, Menéndez Pidal s/n, 14004 Córdoba, Spain; palomaresje@ias.csic.es (J.E.P.-R.); iclavero@ias.csic.es (I.C.-C.); ccantalapiedra@ias.csic.es (C.C.-N.); gleon@ias.csic.es (G.L.-R.); 2 Helmholtz Centre for Environmental Research—UFZ, Department of Ecological Modelling, Permoserstrasse 15, 04318 Leipzig, Germany; antonio.archidona-yuste@ufz.de; 3 Nematology and Chemistry Units, Agricultural Research Organization (ARO), The Volcani Center, Department of Entomology, Bet Dagan 50250, Israel; sigalhor@volcani.agri.gov.il; 4 Nematology Research Unit, Department of Biology, Ghent University, K.L. Ledeganckstraat 35, 9000 Ghent, Belgium; gerrit.karssen@ugent.be; 5 National Plant Protection Organization, Wageningen Nematode Collection, P.O. Box 9102, 6700 HC Wageningen, The Netherlands

**Keywords:** Bayesian inference, cytochrome c oxidase subunit 1, distribution, *D2-D3* expansion domains of *28S* rRNA gene, *ITS1*, phylogeny

## Abstract

Reniform nematodes of the genus *Rotylenchulus* are semi-endoparasites of numerous herbaceous and woody plant roots that occur largely in regions with temperate, subtropical, and tropical climates. In this study, we compared 12 populations of *Rotylenchulus* *borealis* and 16 populations of *Rotylenchulus* *macrosoma*, including paratypes deposited in nematode collections, confirming that morphological characters between both nematode species do not support their separation. In addition, analysis of molecular markers using nuclear ribosomal DNA (*28S*, *ITS1*) and mitochondrial DNA (*coxI*) genes, as well as phylogenetic approaches, confirmed the synonymy of *R. macrosoma* with *R. borealis*. This study also demonstrated that *R. borealis* (= *macrosoma*) from Israel has two distinct rRNA gene types in the genome, specifically the two types of *D2-D3* (A and B). We provide a global geographical distribution of the genus *Rotylenchulus*. The two major pathogenic species (*Rotylenchulus* *reniformis* and *Rotylenchulus* *parvus*) showed their close relationship with warmer areas with high annual mean temperature, maximum temperature of the warmest month, and minimum temperature of the coldest month. The present study confirms the extraordinary morphological and molecular diversity of *R. borealis* in Europe, Africa, and the Middle East and comprises a paradigmatic example of remarkable flexibility of ecological requirements within reniform nematodes.

## 1. Introduction

Reniform nematodes of the genus *Rotylenchulus* are an economically important polyphagous group of highly adapted obligate plant parasites that parasitize numerous plants and crops usually associated with temperate, subtropical, and tropical climates [1]. The genus *Rotylenchulus* Linford and Oliveira [2] comprise 11 valid species; some of them are distributed worldwide, whereas others have shown a limited distribution [1,3,4]. This genus has been reported in 77 countries of Africa, Asia, Europe, North and South America, and Australia [1,3,4]. The influence of future global climate change could shorten the life cycle of these nematodes and may expand the distribution of well-adapted species to drought conditions [5,6]. However, other factors such as the low population density in soil, no apparent harvest losses in some crops, or the difficulties for an accurate identification for some *Rotylenchulus* species could thwart their precise geographical distribution. For these reasons, *Rotylenchulus* spp. could be regarded as a “neglected pathogen”, but also as a potentially dangerous pathogen in the future because of new ecological conditions predicted in global climate change scenarios [7]. Consequently, an updating of the global distribution of this group of nematodes allowed us to know the climatic conditions adapted to each species, which are essential to predict the response of this genus to climate change [8,9].

*Rotylenchulus* spp. show high intraspecific variability of some morphological diagnostic features in immature females (the developmental stage usually employed for species identification) [3], and for this reason, it is necessary to use molecular markers for precise species identification. In this regard, the use of rRNA markers is challenging due to the previously noted presence of several gene copies that are not well homogenized in the genome, and for this reason, several different amplicon sizes and associated sequences can be observed [4]. A prominent example of this high intraspecific variability was established in the study on several populations of *Rotylenchulus macrosoma* by Dasgupta et al. [10] and *R. borealis* by Loof and Oostenbrink [11].

In 1952, Oostenbrink found a population of reniform-shaped nematode in a soil sample from Arnhem (The Netherlands). Subsequent examination and comparison with published descriptions showed that the new nematode represented an undescribed species, proposed as *Rotylenchulus borealis* Loof & Oostenbrink [11], referring to its occurrence in northern countries, since the other species of the genus were mainly known from the tropical and subtropical regions [11]. Some years later, Dasgupta et al. [10] revised the genus *Rotylenchulus* and described a new species from olive in Hulda, Israel, closely related to *R. borealis*, named *Rotylenchulus macrosoma* (original spelling *macrosomus). R. macrosoma* differed from the former by its larger body length of immature females and males (0.52–0.64 mm, 0.50–0.68 mm vs. 0.37–0.46 mm, 0.40–0.49 mm in *R. borealis*, respectively), larger female stylet (18–22 vs. 13–16 μm in *R. borealis*), and longer hyaline portion of immature female tail (h = 13–18 vs. 9–13 μm in *R. borealis*). These limited differences between both species have been confirmed by posterior morphometrics of several African populations studied by Germani [12], as well as the recent *R. macrosoma* populations studied from Europe [3,9].

In 2003, Castillo et al. [13] detected a population of reniform nematodes infecting the roots of wild olive trees (*Olea europea* L. ssp. *sylvestris*) on a sandy soil in Cádiz province, southern Spain, which was identified as *R. macrosoma*. Morphometric of the Spanish population agreed with the original description of *R. macrosoma*, except for a shorter stylet length (15–18 *vs*. 18–22 μm), which was considered as an intraspecific variability. Later on, in 2016, Van den Berg et al. [3] provided morphological and molecular characterization of 6 out of 11 presently known species of *Rotylenchulus*, including three Spanish populations (two and one from Cádiz and Seville provinces, respectively) of *R. macrosoma*, which formed a separate and well-supported clade within phylogenetic trees of *D2-D3* expansion segments of *28S* rRNA, *ITS*, and *hsp90* genes [3]. This study also reported high levels of intraspecific and intra-individual variations of rRNA with two or more distinct types of rRNA genes, namely, type A and B [3]. These phylogenetic relationships were confirmed by posterior studies on additional new reports of *R. macrosoma* populations from several European countries including the Czech Republic, France, Germany, Greece, Hungary, Italy, Portugal, Romania, Serbia, and Spain [4,9]. In a recent study on the integrative characterization of plant-parasitic nematodes of potato in Rwanda, Niragire [14] provided morphological and molecular data of a population of *R. macrosoma* from Burera (North Rwanda), but no sequences were deposited in the National Center for Biotechnology Information (NCBI) database. Molecular data available for *R. borealis* is a *28S* rRNA sequence obtained from a Belgian population (MK558206) and the mentioned sequence for Burera clustered together with the Spanish *R. macrosoma* populations [14]. However, this Belgian population (Oudenaarde, Belgium) of *R. borealis* was not mentioned in the associated paper with the NCBI sequence and no morphological data were available alongside it [15]. This sequence has a 99.45% identity with *R. macrosoma*-KT003748 from Spain. Recently, Qing et al. [16] studied the rRNA variation (intragenomic polymorphism) across 30 terrestrial nematode species and sequenced *28S* and *ITS1* from a population of *R. macrosoma* in Israel, which clustered together in the same clade with *R. macrosoma* populations from Spain and Crete (Greece) and clearly separated from other *Rotylenchulus* spp. Finally, in the last months, one new *28S* rRNA sequence of *R. borealis* from New Delhi, India, was deposited on the NCBI database, MT775429 (95% identity with *R. macrosoma* KT003748 from Spain and 94% identity with *R. borealis* MK558206 from Belgium). All these concerns prompted us to carry out an integrative taxonomic analysis of *R. borealis* from the Netherlands in order to confirm the validity of these species or their synonymization with *R. macrosoma*.

The objectives of this study were (1) to morphometrically and molecularly characterize several populations of *R. macrosoma* from Europe and a population of *R. borealis* from the Netherlands, as well as paratypes of both species deposited in Nematode Collections, and to compare them with previous records; (2) to study the phylogenetic relationships of the European and Dutch populations of *R. macrosoma* and *R. borealis* and compare them with available sequenced populations of these species to establish their validity; and (3) to provide a clear view of the global distribution and the current climatic conditions that affect the distribution of species within the genus *Rotylenchulus.*

## 2. Results

### 2.1. Morphometric Comparison of Paratypes and Several Populations of Rotylenchulus Borealis and Rotylenchulus Macrosoma

**Figure 1:**
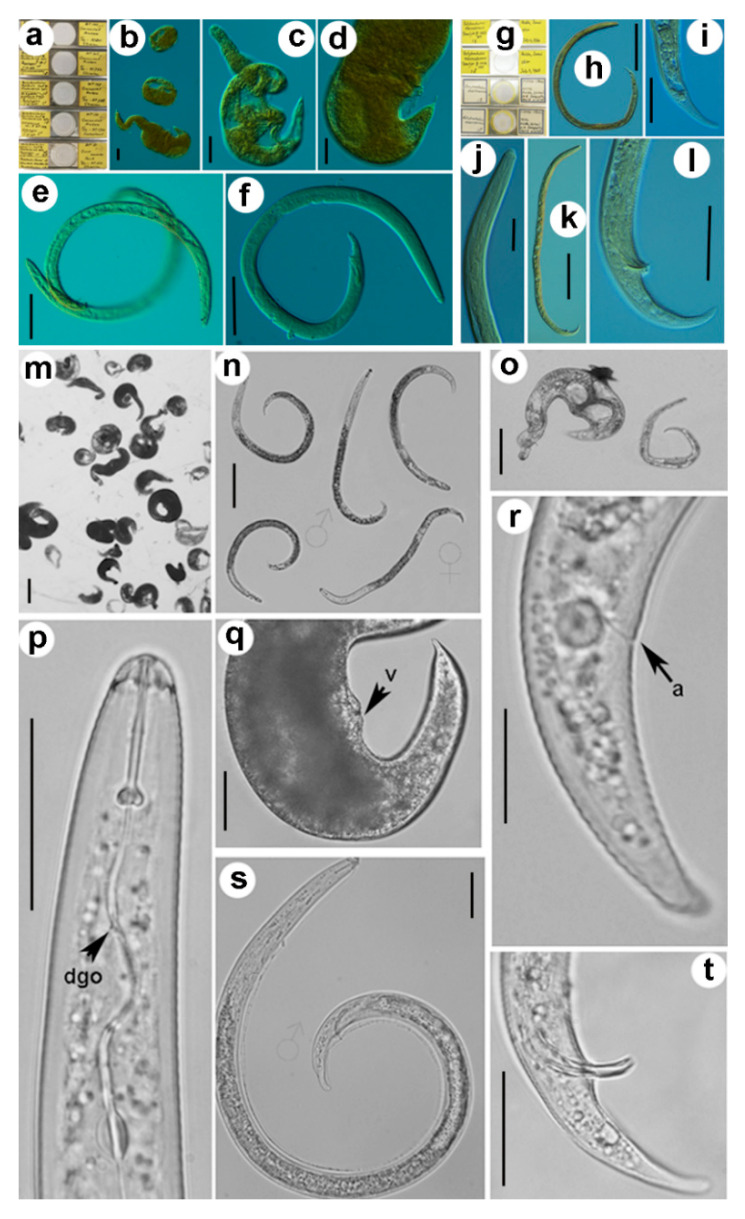
Comparative morphology among paratype specimens of *Rotylenchulus borealis* from the Netherlands (**a**–**f**), paratype specimens of *Rotylenchulus macrosoma* from Israel (**g**–**l**), and a population of *Rotylenchulus macrosoma* from Hungary (**m**–**t**). (**a**,**g**) slides deposited in Wageningen Nematode Collection (WANECO) and United States Department of Agriculture (USDA) nematode collections; (**b**–**d**,**m**,**o**,**q**) mature females; (**e**, **h**–**j**,**n**,**p**,**r**) immature females; (**f**,**k**,**l**,**n**,**s**,**t**) = males. Abbreviations: a = anus; dgo = dorsal gland opening; V = vulva. Scale bars: (**b**–**d**,**h**,**k**,**m**–**o**) 100 μm; (**e**,**f**) 50 μm; (**i**,**j**,**l**,**p**,**q**,**s**,**t**) 20 μm; (**r**) 10 μm.

**Figure 2:**
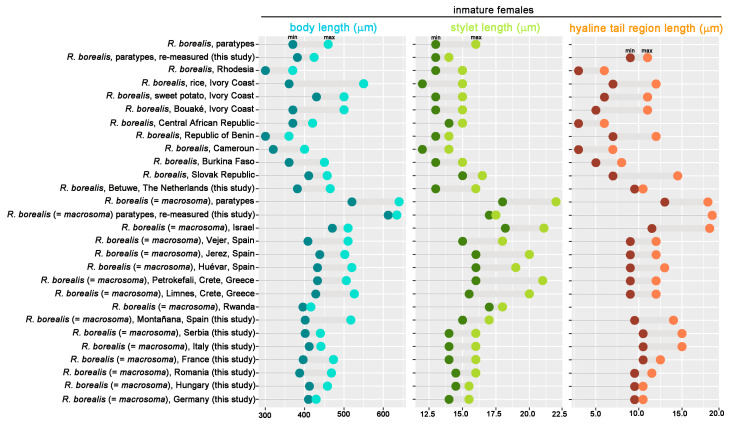
Range (minimum and maximum) comparative key diagnostic measures of immature females (body, stylet, and hyaline female tail lengths) for separating among *R. borealis* and *R. macrosoma* populations in decreasing chronological order of publication.

We detected similar morphological traits in the comparison of 12 populations of *R. borealis* and 16 populations of *R. macrosoma* (Figure 1, Table 1, Table 2, Table 3, Table 4, Table 5, Table 6, Table 7 and Table 8), but ordinary morphometric differences among both species grouped within the three main diagnostic characters of immature females originally used for separating both species (namely, body length, stylet length, and hyaline tail region length) (Figure 2), being the major differences in the original species descriptions. Our data indicated that mean body length of all 12 populations of *R. borealis* was 401.7 μm, whereas the mean for 16 populations of *R. macrosoma* was 483.0 μm. Similarly, stylet and hyaline tail region lengths were 14.25 μm, 7.8 μm vs. 17.28 μm, 12.5 μm, respectively (Table 2, Table 3, Table 4, Table 5, Table 6, Table 7 and Table 8). No differences were detected between the paratype immature females and males of *R. borealis* and the original description, as well as the new studied population from Huissen, Betuwe region (close to the type locality), the Netherlands (Table 2). However, of the two paratype immature females of *R. macrosoma* examined from Wageningen Nematode Collection (WANECO) and United States Department of Agriculture (USDA) nematode collections, both specimens showed a stylet length slightly lower than 18.0 μm (Table 5), and representing a lower measure to that provided in the type population from olive at Hulda, Israel, and quite close to several European populations, such as Spanish populations from Jerez and Huévar del Aljarafe, Cretan populations from Petrokefali and Limnes, or the Rwandan population from Burera. Nevertheless, immature female body and hyaline tail region lengths were similar to those provided in the original description.

### 2.2. Molecular Characterisation and Phylogenetic Analysis of Rotylenchulus Borealis and Rotylenchulus Macrosoma Populations

The amplification of *D2-D3* expansion domains of *28S* rRNA, *ITS1* rRNA, and *coxI* genes of *R. borealis* and *R. macrosoma* populations yielded single fragments of ≈900 bp, 1100 bp, and 450 bp, respectively, on the basis of gel electrophoresis and, in the case of the Israel population, from cloning of the PCR product. Sixteen new sequences from the *D2-D3* of *28S* rRNA gene and eight new sequences from ITS1 rRNA gene were obtained in this study (7 and 9, and 4 and 4, from the Netherlands and Israel, respectively). Four new *coxI* sequences from the Netherlands were deposited in GenBank; however, due to lack of material, it was not possible to obtain *coxI* sequences from Israel. Type B-D2D3 sequence of *R. macrosoma* from Israel was obtained for the first time in this study (MW173975). *D2-D3* for *R. borealis* (MW173970-MW173976) showed a low intraspecific variability with 1–5 different nucleotides and 0 indels (99% similarity). Similarly, intraspecific variability for *D2-D3* in *R. macrosoma* from Israel was slightly higher, with 6–17 different nucleotides and 0–2 indels (97–99% similarity). The molecular diversity of this marker between *R. borealis* (MW173970-MW173976) from the Netherlands and *R. macrosoma* (MW173977-MW173985) from Israel populations was also low, with 5–22 different nucleotides and 0–2 indels (96–99% similarity). *D2-D3* sequences of *R. macrosoma* from Israel (MW173977-MW173985) differed in 0–10 nucleotides and 0 indels (99% similarity) when compared with sequences of *R. macrosoma* deposited in the NCBI database from Spain, Belgium, Serbia, Romania, Hungary, and Portugal, and with *Rotylenchulus* sp. 191_7 (MK558208) and *R. borealis* (MT775429) from Ethiopia and New Delhi in 32, 44 bp, 0, 1 indels (95%, 94% similarity), respectively. Similarly, *D2-D3* sequences of *R. borealis* from the Netherlands (MW173970-MW173976) differed in 14–21 nucleotides and 0 indels (97–98% similarity) when compared with sequences of *R. macrosoma* deposited in the NCBI database from Spain, Belgium, Serbia, Romania, Hungary, and Portugal, and with *Rotylenchulus* sp. 191_7 (MK558208) and *R. borealis* (MT775429) from Ethiopia and New Delhi in 41, 39 bp, 0 indels (94%, 94% similarity), respectively.

The *ITS1* region showed a low intraspecific variability for *R. borealis* (MW174239-MW174242) from the Netherlands, with 0–6 different nucleotides and 0–1 indels (98–100% similarity). Similarly, intraspecific variability for *ITS1* in *R. macrosoma* from Israel (MW174243-MW174246) was low, with 0–11 different nucleotides and 0–4 indels (98–100% similarity). The molecular diversity of this marker between *R. borealis* from the Netherlands (MW174239-MW174242) and *R. macrosoma* from Israel (MW174243-MW174246) populations was also low, with 0–24 different nucleotides and 0–12 indels (95–100% similarity). *ITS1* sequences of *R. macrosoma* from Israel (MW174243-MW174246) differed in 19–32 nucleotides and 1–8 indels (94–96% similarity) when compared with sequences of *R. macrosoma* deposited in the NCBI database from Spain and Greece, and with *Rotylenchulus reniformis* (KF999979) from Japan in 92 bp, 26 indels (86% similarity). Similarly, *ITS1* sequences of *R. borealis* from the Netherlands (MW174239-MW174242) differed in 13–42 nucleotides and 1–11 indels (94–98% similarity) when compared with sequences of *R. macrosoma* deposited in the NCBI database from Spain and Greece, and with *R. reniformis* (KP018567) from China in 137 bp, 54 indels (83% similarity).

The partial *coxI* gene for *R. borealis* from the Netherlands (MW182432-MW182435) showed a low intraspecific variability with 0–8 different nucleotides and 0 indels (98–100% similarity). These sequences differed in 0–47 nucleotides and 0 indels (89–100% similarity) with sequences of *R. macrosoma* deposited in the NCBI database from Spain, Serbia, Romania, Hungary, and Greece, and with *Rotylenchulus parvus* (MK558211) from Tanzania in 64 bp, 4 indels (85% similarity). All molecular markers suggest that populations of *R. borealis* from the Netherlands and *R. macrosoma* from Israel are conspecific.

**Figure 3:**
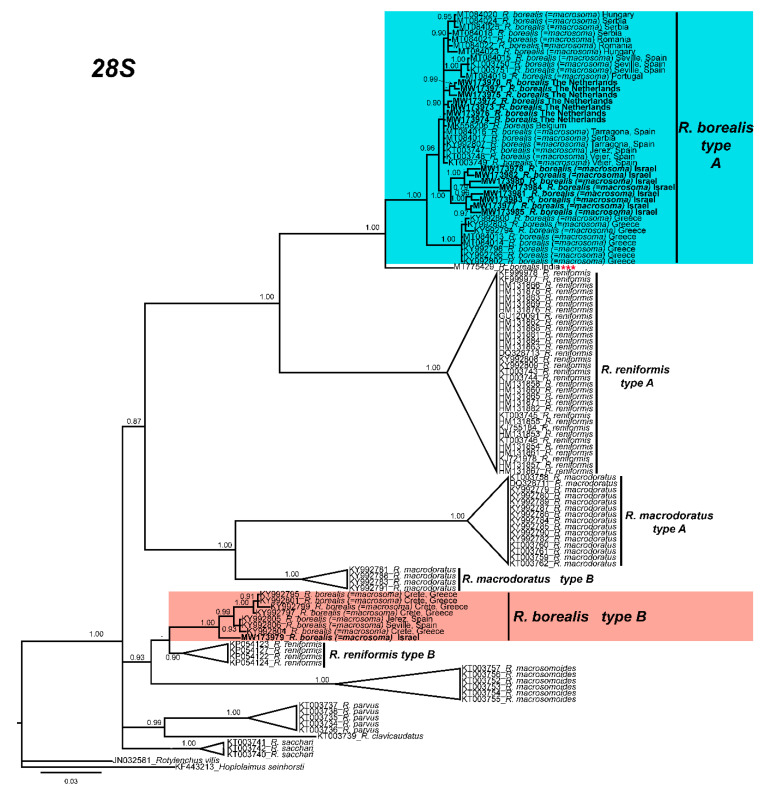
Phylogenetic relationships within the genus *Rotylenchulus*. Bayesian 50% majority rule consensus tree as inferred from D2 and D3 expansion domains of 28S rRNA sequence alignment under the general time-reversible model of sequence evolution with correction for invariable sites and a gamma-shaped distribution (GTR + I + G). Posterior probabilities of more than 0.70 are given for appropriate clades. Newly obtained sequences in this study are shown in bold. Scale bar = expected changes per site. Some branches were collapsed for improving readability of *Rotylenchulus* species. *** Sequence that needs to be revised under integrative taxonomical approaches.

**Figure 4:**
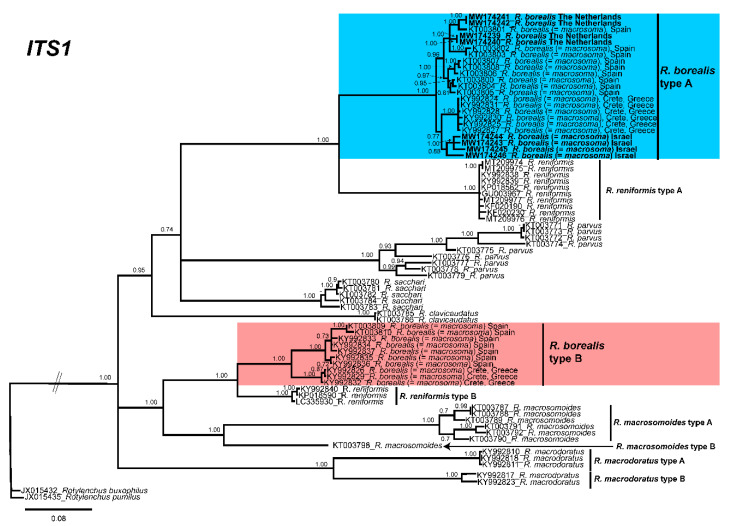
Phylogenetic relationships within the genus *Rotylenchulus*. Bayesian 50% majority rule consensus tree as inferred from *ITS1* rRNA sequence alignment under the general time-reversible model of sequence evolution with correction for invariable sites and a gamma-shaped distribution (GTR + I + G). Posterior probabilities of more than 0.70 are given for appropriate clades. Newly obtained sequences in this study are shown in bold. Scale bar = expected changes per site.

**Figure 5:**
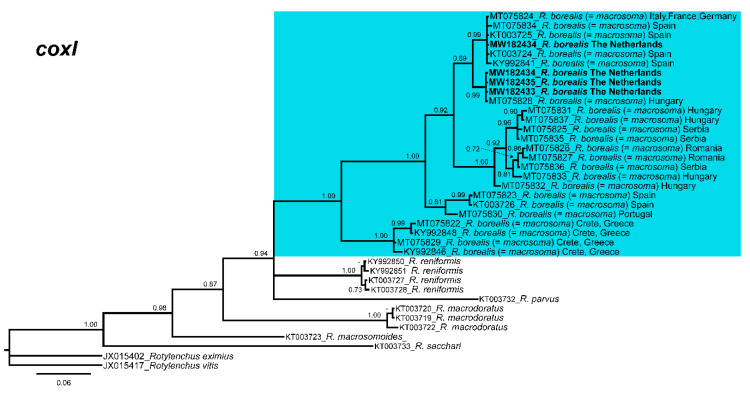
Phylogenetic relationships within the genus *Rotylenchulus*. Bayesian 50% majority rule consensus tree as inferred from *coxI* mitochondrial DNA (mtDNA) sequence alignment under the general time-reversible model of sequence evolution with correction for invariable sites and a gamma-shaped distribution (GTR + I + G). Posterior probabilities of more than 0.70 are given for appropriate clades. Newly obtained sequences in this study are shown in bold. Scale bar = expected changes per site.

Phylogenetic relationships among *Rotylenchulus* species inferred from analyses of *D2-D3* expansion domains of *28S* rRNA, *ITS1*, and partial *coxI* gene sequences using Bayesian inference (BI) are shown in Figure 3, Figure 4 and Figure 5, respectively. The phylogenetic trees generated with the two nuclear and the mitochondrial markers included 123, 77, and 38 sequences, with 704, 888, and 355 positions in length, respectively (Figure 3, Figure 4 and Figure 5). *D2-D3* tree of *Rotylenchulus* spp. showed two moderately supported clades including *R. borealis* type A and type B sequences (posterior probabilities (PP) = 0.87, 0.93, respectively), including *R. reniformis*, *Rotylenchulus macrodoratus*, and *Rotylenchulus macrosomoides* (Figure 3). All sequences of *R. borealis* from the Netherlands (MW173970-MW173976) and Belgium (MK558206), as well as those of *R. borealis* (= *R. macrosoma*) from Israel and all the sequences from Spain, Serbia, Romania, Hungary, and Greece deposited in the NCBI database clustered together in a highly supported clade (PP = 1.00) and were well separated (PP = 1.00) from *28S* of *R. borealis* (MT775429) from New Delhi (Figure 3).

The 50% majority rule consensus *ITS1* BI tree also showed two clades, one moderately and the other well supported including *R. borealis* type A and type B sequences (PP = 0.95, 1.00, respectively), including *R. reniformis*, *R. parvus*, *Rotylenchulus sacchari*, and *Rotylenchulus clavicaudatus* (Figure 4). All sequences of *R. borealis* from the Netherlands (MW174239-MW174242) and those of *R. borealis* (= *macrosoma*) from Israel and all the sequences from Spain and Greece deposited in the NCBI database clustered together in a highly supported clade (PP = 1.00).

Finally, the phylogenetic relationships of *Rotylenchulus* species inferred from analysis of partial *coxI* gene sequences showed several clades that were well defined (Figure 5). All sequences of *R. borealis* from the Netherlands (MW182432-MW182435) and sequences from several European countries (Germany, Greece, Hungary, Italy, Portugal, Romania, Serbia, and Spain) deposited in the NCBI database clustered together in a highly supported clade (PP = 1.00).

### 2.3. Global Distribution Rotylenchulus spp.

**Figure 6:**
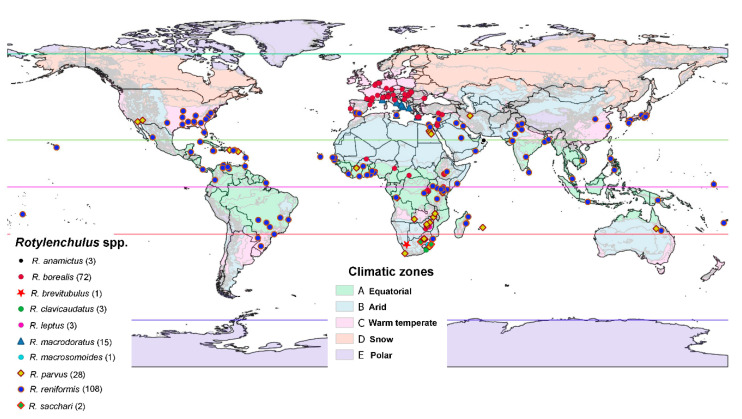
World map distribution of *Rotylenchulus* species across different climate conditions. Climatic zones based on type of vegetation [19]. In the species list, the number in brackets indicates the locations cited for each species.

**Figure 7:**
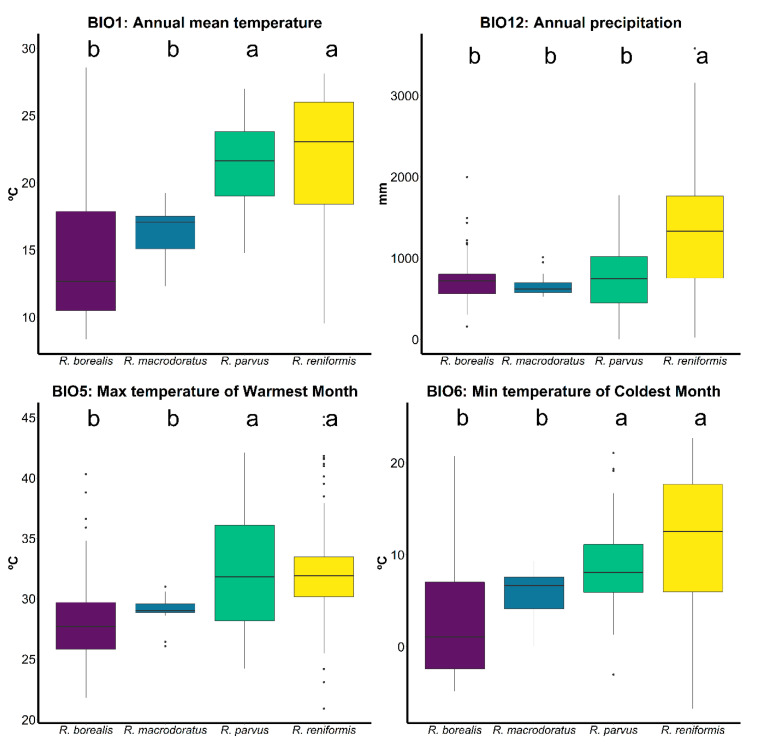
Annual mean temperature, annual precipitation, maximum temperature of warmest month, and minimum temperature of coldest month for *Rotylenchulus* species with ≥ 3 reports (each single dot correspond to a species report). The different lowercase letters indicate the differences in each bioclimatic variable between species. They were tested using ANOVA with a level of significance of *p* < 0.05.

We detected that the genus *Rotylenchulus* exhibited a worldwide distribution across a wide variety of environments and climatic zones (Figure 6). We found that *Rotylenchulus* spp. are widely distributed in warm temperature (−3 °C < annual mean temperature < +18 °C) and arid (annual precipitation < 300 mm) climate zones, with seven different species for both types, and to a lesser extent in equatorial (annual mean temperature ≥ +18 °C) and snow (mean temperature of the coldest month ≤ −3 °C) climate zones, with four and one species, respectively (Figure 6). We did not detect species in the polar (mean temperature of the warmest month < +10 °C) climate zone (Figure 6). It should be noted that highest diversity of species, although less frequently found, seems to be in the southern part of Africa with mainly warm temperate and arid climatic zones (Figure 6). The species distribution observed in this study revealed that the genus *Rotylenchulus* is adapted to heterogeneous climatic conditions, with an annual mean temperature of 19.14 °C, but ranging from 8.36 to 28.58 °C, and a mean annual precipitation of 1026.97 mm, but ranging from 1 to 3583.00 mm. This suggests that the occurrence of *Rotylenchulus* species in areas with extremely low values in annual precipitation (i.e., desert lands in Egypt and Iraq; Figure 6) could be due to the establishment of an irrigation regime in agricultural ecosystems. Only four species were reported more than three times in literature review, i.e., *R. borealis* (= *R. macrosoma*), *R. macrodoratus*, *R. parvus*, and *R. reniformis* (Figure 6). The most widely distributed species was *R. reniformis*, followed by *R. parvus*, both reported in all continents except Antarctica (Africa, North and South America, Asia, Australia, and Europe), and *R. borealis* in Africa, Europe, and Middle East Asia (Figure 6). Bioclimatic variables (BIOCLIM) based on temperature (annual mean temperature (BIO1), maximum temperature of warmest month (BIO5), and minimum temperature of coldest month (BIO6)) showed significantly different temperature conditions on the distribution of these most common species (Figure 7). The two major pathogenic species (*R. reniformis* and *R. parvus*) were mainly distributed in tropical, temperate, and arid climates, showing their close relationship with warmer areas with high annual mean temperature, max temperature of the warmest month, and minimum temperature of the coldest month, ranging from 9.55 to 21.11 °C, 24.00 to 3583.00 mm and 14.79 to 26.99 °C, 1.00 to 1773.00 mm, respectively (Figure 6 and Figure 7). *Rotylenchulus macrodoratus* showed a distribution in temperate climate with annual mean temperature and precipitation ranging from 12.32 to 19.23 °C and 526.00 to 1013.00 mm, respectively (Figure 7). The climatic plasticity of *R. borealis* is remarkable in relationship with annual mean temperature and precipitation, ranging from 8.36 to 28.58 °C and 160.00 to 1998.00 mm, respectively (Figure 7). *Rotylenchulus borealis* (= *R. macrosoma*) showed statistically significant differences in lower annual mean temperature, max temperature of the warmest month, and min temperature of the coldest month in comparison to *R. parvus* and *R. reniformis* (Figure 7). However, only *R. reniformis* showed statistically significant differences in higher annual precipitation in comparison to the other studied species (Figure 7). Other bioclimatic variables are shown in Figure S1.

## 3. Discussion

The primary objective of this study was to decipher the intraspecific diversity of *R. borealis* and *R. macrosoma* by applying integrative taxonomical approaches on several new unidentified *Rotylenchulus* populations from Europe, appearing morphological and morphometrically undistinguishable. Additionally, we aimed to provide new insights into the global distribution and climatic requirements of the genus *Rotylenchulus*.

The resemblance between the mature females of *R. borealis* and *R. macrosoma*, as well as the general similarity between these two species also in their male and immature female forms, host preferences, and host tissue reactions was emphasized by Cohn and Mordechai [18] studying a topotype population of *R. macrosoma* from olive under growth chamber conditions. Our morphometric studies in this research support that both species do not have major differences in basic morphology or in morphometric informative characters such as immature female body length, stylet length, tail hyaline region, and spicules morphology and morphometry, showing a remarkable example of a close phylogenetic relationship of both species. The results on our new measurements on *R. macrosoma* immature female paratype specimens from WANECO and USDA nematode collections suggest that the range in stylet length could probably be shorter than that provided in the original description [10], but unfortunately no other paratypes could be studied. The morphometric comparison of an important number of populations from *R. borealis* and *R. macrosoma* exhibited morphometric variation normally expected among populations of the same *Rotylenchulus* species. The higher values in all of the three main distinguishing morphometric characters between both species were detected in Israel, Crete, and a Spanish population from Huévar del Aljarafe (southern Spain), but these differences do not justify the separation in two different species [3,4,9,10].

In the present study, in which sequence data obtained from *28S* and *ITS1* rRNA genes and *coxI* mitochondrial DNA (mtDNA) gene was analyzed, specimens from populations identified as representing *R. borealis* and *R. macrosoma* from the Netherlands and several European countries, including Israel, respectively, clustered together as a single group. This grouping was well supported by the high bootstrap values in the phylogenetic analysis, thereby supporting the synonymization of *R. macrosoma* with *R. borealis*, as already emphasized by Cohn and Mordechai [18].

Phylogenetic analyses based on three molecular markers (*D2-D3* expansion domains of *28S* rRNA gene, *ITS1* region, and the partial *coxI* mtDNA) resulted in a general consensus of species phylogenetic positions clustering *R. borealis* population from the Netherlands with *R. macrosoma* from Israel, together with all other *R. macrosoma* populations previously reported in several European countries. These phylogenetic analyses were congruent with those given by previous studies [3,4,9,16,20], and phylogeny of the *28S* rRNA and ITS regions confirm that *R. borealis* population from the Netherlands is conspecific with *R. macrosoma* from Israel and all other populations from Europe. Our results on *28S* rRNA phylogeny also suggest that *R. borealis* (MT775429) from New Delhi could not be considered conspecific with *R. borealis* and needs to be revised under integrative taxonomical approaches for confirming its specific status. The genus *Rotylenchulus* has rRNA genes that exhibit high levels of intraspecific and intra-individual variation [3,9,16]. However, they seem functional through the reconstruction of secondary structure models and mutation mapping using *R. reniformis* sequences [3]. Qing et al. [16] suggested that these different sequences are paralogs located in different rRNA clusters or chromosomes and that these tandem arrays may still be expanding in number.

Longer stylet specimens do not seem to be associated with differences in molecular markers (as some Andalusian populations with longer stylet were molecularly associated with other species with shorter stylets) (Figure 3, Figure 4 and Figure 5). Other characters (body length and hyaline tail region length), as shown in Figure 2, seem to be very variable for African populations of *R. borealis*. Palomares-Rius et al. [4], in a broad molecular study of *R. borealis* (*= R. macrosoma*), also studied the molecular species separation, with the results showing incongruent results for species separation between Cretan and other European populations for *R. borealis* (*= R. macrosoma*), even with the relatively high molecular differences between both population groups. In our case, the new population of *R. borealis* found in the Netherlands in this study, and the sequence deposited in GenBank from Belgium (MK558206), had an even lower molecular similarity with other former *R. macrosoma* populations from Crete, Greece, fully supporting our idea of conspecificity.

Thus, the morphological and morphometric results of both species groups, together with the high molecular similarity among ribosomal and mitochondrial genes of both species groups, do not support the validity of *R. macrosoma* as a separate species and give sufficient basis for the synonymization of *R. macrosoma* n. syn. with *R. borealis*. Since the description of *R. borealis* was in 1962 and that of *R. macrosoma* in 1968, the name *R. borealis* has priority over *R. macrosoma*; thus, *R. macrosoma* is proposed here as a junior synonym of the former.

Climate is a critical environmental determinant of the distribution of plant-parasitic nematodes and a key driver of their reproduction and survival [21]. Temperature, moisture, and availability of host plants are three of the most important factors governing the distribution, spread, and symptom development in plants from plant-parasitic nematodes [21,22], including reniform nematodes. The wide distribution of *Rotylenchulus* species likely resulted from an exceptionally wide host range, as well as their ability to survive extended periods in a dehydrated state [1]. Anhydrobiotic *Rotylenchulus* forms have been documented dispersing long distances in dust storms [23]; however, human dispersion through agriculture activities need also to be considered [4]. The influence of annual precipitation on *Rotylenchulus* spp. distribution suggests that this factor may be not as important as expected. However, the majority of the recorded points have crops with irrigation, and this could change the natural precipitation conditions and importance for these species. In particular, the widespread presence of *R. borealis* in localities at higher latitude in Northern Europe and lower latitude in several central African countries indicated and adaptation to heterogeneous climatic conditions and probably survival strategies for colder and warmer winters and humid to dry soil conditions. Similarly, the cosmopolitan distribution of *R. parvus* can be related to the wide range of temperature reproduction (20 to 35 °C) and survival (4 to 35 °C), as suggested by Dasgupta and Raski [24]. Climate change could expand *R. borealis* to upper latitudes as climate will warm and this will fulfil the ecological requirements of this species, one of the most adapted to lower temperatures among the four most distributed species (*R. borealis*, *R. macrodoratus*, *R. parvus*, and *R. reniformis*). Interestingly, the major diversity of the genus is from sub-Saharan Africa, with the exception of *R. macrodoratus* (Mediterranean distribution) and *R. leptus* (Arabian Peninsula). Siddiqi [25] proposed the idea about the origin of this genus in the Afrotropical (Ethiopian) zoogeographical region, comprising Africa (south of the Sahara); the southern part of the Arabian Peninsula; and various islands, including Madagascar. This idea was reinforced with phylogenetic analysis [3]. However, only three species (*R. borealis*, *R. parvus*, and *R. reniformis*) have been able to colonize different continents with wide ecological requirements, as was shown in this research. Additionally, to these ecological requirements for species distribution, other factors such as survival in anhydrobiotic stage or resting eggs could help with the dispersal of this species to other agricultural areas in the world.

In summary, the present study confirmed the synonymy of *R. macrosoma* with *R. borealis*, and thus the genus comprises 10 valid species. Our data also demonstrate the extraordinary morphological and molecular diversity of *R. borealis* in Europe, Africa, and the Middle East and comprise a paradigmatic example of remarkable flexibility of climatic requirements within reniform nematodes. Nevertheless, despite frequent surveys in different continents of the world, the number of sites studied is still low. Therefore, further surveys are still needed in unsampled geographical areas and climatic conditions, both in plantations and indigenous forests with the aim to identify additional *Rotylenchulus* species.

## 4. Materials and Methods

### 4.1. Nematode Populations and Morphometric Studies

One of the authors (G. Karssen) visited the type locality of *R. borealis*, and the place reported in the original description was lost, i.e., was filled up by new building of houses. Nevertheless, this author detected a new population of *R. borealis* in another location close near the type locality, at Huissen, Betuwe region, the Netherlands. This new population, together with mounted paratypes from of *R. macrosoma* and *R. borealis* from the nematode collections Wageningen Nematode Collection (WANECO; slides WT106, WT107, WT110, WT111, and #1025 NT and #1026 NT) and USDA Nematode Collection kindly provided by Dr. Z. A. Handoo (slides T-594p and T-595p), were used for morphological studies.

In addition, some new European reports recently detected and associated with corn and wheat [4] were measured in order to carry out a morphometric comparison with all the measured populations of both species (Table 2, Table 3, Table 4, Table 5, Table 6, Table 7 and Table 8). All these populations were compared with the morphometry of all previously studied populations of both species, including a total of 12 populations of *R. borealis* and 16 populations of *R. macrosoma*.

Nematodes were extracted from 500 cm^3^ of soil by centrifugal flotation [26] method. For morphometric studies, *Rotylenchulus* specimens were killed and fixed by a hot solution of 4% formalin + 1% glycerol, then processed in pure glycerin [27], as modified by De Grisse [28]. The light micrographs and measurements of each nematode population including important diagnostic characteristics (i.e., de Man indices, body length, stylet length, lip region, tail length, etc.) were performed using a Leica DM6 compound microscope with a Leica DFC7000 T digital camera. Nematodes were identified at the species level using an integrative approach combining molecular and morphological techniques to achieve efficient and accurate identification [4,9]. For each nematode population, key diagnostic characters were determined, including body length, stylet length, a ratio (body length/maximum body width), c’ ratio (tail length/body width at anus), V ratio ((distance from anterior end to vulva/body length) × 100), and o ratio ((distance from stylet base to dorsal pharyngeal opening/body length) × 100) [9], and the sequencing of specific DNA fragments (described below) confirmed the identity of the nematode species for each population.

### 4.2. DNA Extraction, PCR, and Sequencing

For molecular analyses, in order to ensure that the selected nematodes for extracting DNA are from the same species, we temporary mounted 2 live nematodes from each sample in a drop of 1M NaCl containing glass beads (to avoid nematode crushing/damaging specimens) to ensure specimens conformed to the unidentified populations of *Rotylenchulus*. All necessary morphological and morphometric data by taking pictures and measurements using the above camera-equipped microscope were recorded. This was followed by DNA extraction from a single specimen and polymerase chain reaction (PCR) cycle conditions, as previously described [4,9]. PCR and sequencing of the Dutch population was performed at the Institute for Sustainable Agriculture, Spanish National Research Council (IAS-CSIC) facility, whereas for the Israeli population at Agricultural Research organization (ARO)-Volcani Center, Israel. Several sets of primers were used for PCR. A partial region of the *28S* rRNA gene including the expansion domains D2 and D3 (*D2-D3*) was amplified by using the primers D2A (5′-ACAAGTACCGTGAGGGAAAGTTG-3′) and D3B (5′-TCGGAAGGAACCAGCTACTA-3′) [29]. The internal transcribed spacer region (*ITS*) was amplified using forward primer TW81 (5′-GTTTCCGTAGGTGAACCTGC -3′) and reverse primer AB28 (5′-ATATGCTTAAGTTCAGCGGGT-3′) [30]. The *coxI* gene was amplified using the primers JB3 (5′-TTTTTTGGGCATCCTGAGGTTTAT-3′) and JB5 (5′-AGCACCTAAACTTAAAACATAATGAAAATG-3′) [31]. The PCR cycling conditions for the *28S* rRNA primers were as follows: 94 °C for 2 min, followed by 35 cycles of 94 °C for 30 s, an annealing temperature of 55 °C for 45 s, and 72 °C for 1 min, and 1 final cycle of 72 °C for 10 min. The PCR cycling for *coxI* primers was as follows: 95 °C for 15 min, 39 cycles at 94 °C for 30 s, 53 °C for 30 s, and 68 °C for 1 min, followed by a final extension at 72 °C for 7 min. PCR volumes were adapted to 25 μL for each reaction, and primer concentrations were as described in De Ley et al. [29] and Bowles et al. [31]. We used 5x HOT FIREpol Blend Master Mix (Solis Biodyne, Tartu, Estonia) in all PCR reactions. The PCR products were purified after amplification using ExoSAP-IT (Affimetrix, USB products, Kandel, Germany) and used for direct sequencing in both directions with the corresponding primers. Israeli amplification products were cloned before sequencing using pGEM-T easy vector systems (Promega). The resulting products were purified and run in a DNA multicapillary sequencer (Model 3130XL Genetic Analyzer; Applied Biosystems, Foster City, CA, USA), using the BigDye Terminator Sequencing Kit v.3.1 (Applied Biosystems) at the Stab Vida sequencing facility (Caparica, Portugal). The sequence chromatograms of the 2 markers (*coxI* and *D2-D3* expansion segments of *28S* rRNA) were analyzed using DNASTAR LASERGENE SeqMan v. 7.1.0. Basic local alignment search tool (BLAST) at the National Center for Biotechnology Information (NCBI) was used to confirm the species identity of the DNA sequences obtained in this study [32]. The newly obtained sequences were deposited in the GenBank database under accession numbers indicated on the phylogenetic trees and in Table 1.

### 4.3. Phylogenetic Analysis

Sequenced genetic markers in the present study (after discarding primer sequences and ambiguously aligned regions) and several *Rotylenchulus* spp. sequences obtained from GenBank were used for phylogenetic reconstruction (Table 1). Outgroup taxa for each dataset were selected on the basis of previous published studies [3,4,9]. Multiple sequence alignments of the newly obtained and published sequences were made using the Fast Fourier transform-normalized similarity matrix (FFT-NS-2) algorithm of MAFFT v. 7.450 [33]. Sequence alignments were visualized using BioEdit [34] and edited by Gblocks ver. 0.91b [35] in Castresana Laboratory server (http://molevol.cmima.csic.es/castresana/Gblocks_server.html) using options for a less stringent selection (minimum number of sequences for a conserved or a flanking position: 50% of the number of sequences + 1; maximum number of contiguous no conserved positions: 8; minimum length of a block: 5; allowed gap positions: with half).

Phylogenetic analyses of the sequence datasets were based on Bayesian inference (BI) using MRBAYES 3.2.7a [36]. The best-fit model of DNA evolution was calculated with the Akaike information criterion (AIC) of JMODELTEST v. 2.1.7 [37]. The best-fit model, the base frequency, the proportion of invariable sites, and the gamma distribution shape parameters and substitution rates in the AIC were then used in phylogenetic analyses. BI analyses were performed under a general time reversible, with a proportion of invariable sites and a rate of variation across sites (GTR + I + G) model for *D2-D3*, *ITS1* rRNA, and the partial *coxI* gene. These BI analyses were run separately per dataset with 4 chains for 2 × 10^6^ generations. The Markov chains were sampled at intervals of 100 generations. Two runs were conducted for each analysis. After discarding burn-in samples of 30% and evaluating convergence, we retained the remaining samples for more in-depth analyses. The topologies were used to generate a 50% majority-rule consensus tree. Posterior probabilities (PP) were given on appropriate clades. Trees from all analyses were visualized using FigTree software version v.1.42 [38].

### 4.4. Data Collection of Global Distribution of Rotylenchulus spp. and Statistical Analysis

The species distribution data of *Rotylenchulus* spp. were exhaustively compiled from the national and regional nematofauna records worldwide from databases (Google Scholar, Web of Sciences, Scopus, and PubMed) and specialized literature (nematological and phytopathological journals) during the period 2020–1940. We selected only those articles satisfying one the following criteria for this review: (1) contained geographical information about the presence and/or abundance of reniform nematodes (*Rotylenchulus* spp.); (2) contained data on their taxonomy, morphology, molecular identification, ecology, pathogenicity, and provided localities of each population. Articles lacking information about geographic coordinates were cross-checked using Quantum GIS v. 3.12.0 [39]. Nevertheless, since *R. reniformis* has been associated with hundreds of crops and native plants in many regions of the world (on the four aforementioned databases we found 9640, 1377, 446, and 189 studies, respectively), only selected reports concerning geographical information were selected, and duplicity of reported localities were not included.

We used bioclimatic predictors (BIOCLIM) based on temperature and precipitation [40] to detect environmental conditions associated with the global distribution of *Rotylenchulus* spp. and to compare the climate spaces for the different species. Additionally, we plotted the global distribution *Rotylenchulus* spp. across climate zones on the basis of the type of vegetation [19]. Only species with more than 3 reported populations were plotted in order to assess the range of climatic variables for each species. Species with type locality only or occasional records were omitted.

The analysis on the bioclimatic variables for *Rotylenchulus* spp. with more than 3 reported populations was concentrated in 18 variables: BIO1 (Annual mean temperature), BIO2 [Mean Diurnal Range (Mean of monthly (max temp-min temp)], BIO3 [Isothermality, (BIO2/BIO7) * 100], BIO4 [Temperature seasonality, (standard deviation * 100)], BIO5 (maximum temperature of the warmest month), BIO6 (minimum temperature of the coldest month), BIO7 [temperature annual range (BIO5-BIO6)], BIO9 (mean temperature of driest quarter), BIO10 (mean temperature of the warmest quarter), BIO 15 (precipitation seasonality, coefficient of variation), and BIO18 (precipitation of the warmest quarter). To detect the influence on *Rotylenchulus* spp. of the different bioclimatic variables, we used one-way ANOVA among species conducted using the R v. 3.5.1 freeware [41]

## Figures and Tables

**Table 1:** Populations sampled for *Rotylenchulus* spp. from two localities in the Netherlands and Israel used in this study.

Locality, Country	Nematode Code	*D2-D3*	*ITS1*	*coxI*
*Rotylenchulus borealis*				
Huissen, Betuwe region (the Netherlands)	AV23	MW173970	MW1742399	MW182432
Huissen, Betuwe region (the Netherlands)	AV25	MW173971	MW174240	-
Huissen, Betuwe region (the Netherlands)	AV26	MW173972	-	MW182433
Huissen, Betuwe region (the Netherlands)	AV27	MW173973	MW174241	MW182434
Huissen, Betuwe region (the Netherlands)	AV28	MW173974	MW174242	MW182435
Huissen, Betuwe region (the Netherlands)	AV29	MW173975	-	
Huissen, Betuwe region (the Netherlands)	AV30	MW173976	-	
*Rotylenchulus macrosoma*				
Beit She’an (Israel)	C26	MW173977	-	-
Beit She’an (Israel)	C27	MW173978	-	-
Beit She’an (Israel)	C29	MW173979	-	-
Beit She’an (Israel)	C30	MW173980	-	-
Beit She’an (Israel)	C31	MW173981	MW174243	-
Beit She’an (Israel)	C32	MW173982	MW174244	-
Beit She’an (Israel)	C45	MW173983	MW174245	-
Beit She’an (Israel)	C46	MW173984	MW174246	-
Beit She’an (Israel)	C47	MW173985	-	-

(-) Not obtained or not performed.

**Table 2:** Measurements of immature females and males of *Rotylenchulus borealis* from type locality in the Netherlands and several localities in Africa. All measurements are in micrometers and in the form mean ± SD (range).

	Grasses, Arnhem, the Netherlands, Paratypes		Paratypes from WANECO Nematode Collection, Re-Measured in This Study		(*R. variabilis* = *R. borealis*) *Rumex* sp., Inyanga Orchard Area, Southern Rhodesia (Dasgupta et al. [10]		Upland Rice, Ferkessedougou, Ivory Coast [12]		Sweet Potato, Ferkessedougou, Ivory Coast [12]	
Character/Ratio	Immature Females	Males	Immature Females	Males	Immature Females	Males	Immature Females	Males	Immature Females	Males
n	20	40	3	5	22	21	18	17	5	8
L ^a^	(370–460)	(400–490)	408 ± 24 (381–424)	418 ± 12 (404–435)	(300–370)	(340–410)	420 (360–550)	480 (400–540)	470 (430–500)	520 (480–570)
a	(22.5–32.5)	(30.3–40.2)	27.9 ± 2.3 (25.4–29.9)	24.1 ± 2.8 (21.6–28.9)	(22–26)	(22–33)	27 (23–31)	29.2 (25–42)	29 (26–31)	28 (24–36)
b	(2.5–3.4)	(3.2–4.0)	4.1 ± 0.2 (3.9–4.2)	3.8 ± 0.2 (3.4–3.9)	(3.3–3.9)	(3.1–4.1)	2.9 (2.0–3.3)	4.4 (3.8–5.2)	3.0 (2.8–3.6)	4.5 (4.5–5.0)
c	(11.3–14.8)	(12.1–15.8)	14.9 ± 0.5 (14.6–15.5)	14.7 ± 1.0 (13.6–16.1)	(13.0–16.0)	(14–20)	14.7 (12.5–17.3)	16 (14–18)	14.3 (12.0–16.0)	15 (11–18)
c’	-	-	2.8 ± 0.2 (2.6–2.9)	2.8 ± 0.1 (2.6–2.9)	(2.6–3.2)	-	3.4 (3.0–4.0)	-	3.5 (3.1–4.4)	-
V or T	(57.6–64.8)	(25.0–54.0)	62.2 ± 1.0 (61.0–63.0)	39.7 ± 13.5 (23.4–56.4)	(59–66)	(29–51)	61 (57–67)	-	62 (59–66)	-
o	-	-	124.4 ± 3.7 (121.4–128.6)	136.7 ± 8.1 (130.8–150.0)	(120–138)	-	131 (113–160)	-	124 (113–147)	-
DGO	-	-	17.0 ± 1.0 (16.0–18.0)	17.2 ± 0.8 (16.0–18.0)	-	-	-	-	-	-
Stylet length	(13.0–16.0)	(12.0–14.0)	13.7 ± 0.6 (13.0–14.0)	12.6 ± 0.5 (12.0–13.0)	(13.0–15.0)	(10.0–12.0)	14.0 (12.0–15.0)	13.0 (10.0–14.0)	14.5 (13.0–15.0)	14.0 (13.0–14.0)
Lip region width	-	-	6.3 ± 0.6 (6.0–7.0)	6.5 ± 0.5 (6.0–7.0)	-	-	-	-	-	-
Tail length	-	-	27.3 ± 1.5 (26.0–29.0)	28.6 ± 2.3 (26.0–32.0)	-	-	-	-	-	-
h	-	-	10.0 ± 1.0 (9.0–11.0)	9.9 ± 0.7 (9.0–11.0)	(3.0–6.0)	(3.0–7.0)	9.0 (7.0–12.0)	8.0 (6.0–10.0)	8.2 (6.0–11.0)	9.0 (6.0–11.0)
Spicule length	-	(20.0–21.0)	-	21.2 ± 0.8 (20.0–22.0)	-	(19.0–23.0)	-	22.7 (18.0–24.0)	-	23.0 (20.0–26.0)
Gubernaculum length	-	(7.0–8.0)	-	7.0 ± 0.7 (6.0–8.0)	-	(7.0–9.0)	-	9.0 (6.0–10.0)	-	10.0 (8.0–12.0)

^a^ a = body length/maximum body width; b = body length/pharyngeal length; c = body length/tail length; c’ = tail length/body width at anus; DGO = distance from stylet base to dorsal gland opening; h = hyaline tail region length; o = (DGO/stylet length) × 100; V = (distance from anterior end to vulva/body length) × 100.

**Table 3:** Measurements of immature females and males of *Rotylenchulus borealis* from several localities in Africa. All measurements are in micrometers and in the form mean ± SD (range).

	Sweet Potato, Bouaké, Ivory Coast [12]		Upland Rice, Bambari, Central African Republic [12]		Cotton, North Republic of Benin [12]	
Character/Ratio	Immature Females	Males	Immature Females	Males	Immature Females	Males
n	6	2	9	16	6	9
L ^a^	420 (370–500)	500, 520	390 (370–420)	380 (360–410)	330 (300–360)	360 (320–370)
a	25 (24–26)	26, 27	25.2 (23–27)	27 (21–30)	23 (21–26)	24 (21–28)
b	2.9 (2.6–3.4)	5.0, 5.7	2.8 (2.6–3.2)	3.4 (2.7–3.9)	2.7 (2.6–2.9)	3.3 (2.6–3.7)
c	16.7 (13.4–25.5)	14.6, 17.2	15.5 (14.0–17.2)	17 (14–21)	14.2 (12.7–15.4)	15.0 (8.7–16.7)
c’	3.2 (2.9–3.3)	-	2.7 (2.3–3.1)	-	2.7	-
V or T	62 (60–64)	-	63.7 (61–65)	-	63.6 (64–65)	-
o	119 (100–127)	-	112 (100–128)	-	134 (130–143)	-
DGO			6.8 (5.0–8.0)	6.0 (3.0–10.0)	6.0 (5.0–8.0)	-
Stylet length	14.0 (13.0–15.0)	-	15.0 (14.0–15.0)	12.0 (11.0–13.0)	13.0 (13.0–14.0)	6.3 (4.0–8.0)
Lip region width	-	-	-	22.0 (17.0–25.0)	-	19.0 (17.0–22.0)
Tail length	-	-	-	8.0 (7.0–11.0)	-	8.0 (7.0–10.0)
h	7.5 (5.0–11.0)	8.0, 9.0	(3.0–6.0)	(3.0–7.0)	9.0 (7.0–12.0)	8.0 (6.0–10.0)
Spicule length	-	22.0, 24.0	-	(19.0–23.0)	-	22.7 (18.0–24.0)
Gubernaculum length	-	-	-	(7.0–9.0)	-	9.0 (6.0–10.0)

^a^ a = body length/maximum body width; b = body length/pharyngeal length; c = body length/tail length; c’ = tail length/body width at anus; DGO = distance from stylet base to dorsal gland opening; h = hyaline tail region length; o = (DGO/stylet length) × 100; V = (distance from anterior end to vulva/body length) × 100.

**Table 4:** Measurements of immature females and males of *Rotylenchulus borealis* from several localities in Africa and the Slovak Republic. All measurements are in micrometers and in the form mean ± SD (range).

	Cotton, North Cameroon [12]		Peanut, Burkina Faso [12]		Corn, Somotor, Slovak Republic [17]		Grasses, the Netherlands, This Study	
Character/Ratio	Immature Females	Males	Immature Females	Males	Immature Females	Males	Immature Females	Males
n	10	10	15	2	8	5	5	10
L ^a^	370 (320–400)	390 (360–420)	400 (360–450)	480, 490	428 ± 18 (410–457)	445 ± 17 (416–459)	427 ± 361 (381–465)	432 ± 28 (400–490)
a	22.6 (18–29)	25.6 (23–28)	24.5 (21–31)	21, 31	29 ± 2.3 (27.0–34.5)	31 ± 1.5 (28.7–32.1)	27.5 ± 3.9 (22.5–32.5)	29.2 ± 4.3 (25.1–40.2)
b	2.6 (2.3–3.0)	3.1 (2.4–3.6)	2.7 (2.4–3.2)	2.4, 3.2	4.2 ± 0.3 (3.9–4.6)	4.0 ± 0.1 (3.6–3.9)	3.2 ± 0.5 (2.5–3.8)	3.6 ± 0.2 (3.2–4.0)
c	15.5 (12–21)	14.9 (13.5–16.0)	15.8 (13.8–18.1)	17.4, 21.3	13.9 ± 1.4 (12.7–16.7)	14.0 ± 0.1 (12.8–15.3)	13.8 ± 1.6 (11.3–15.0)	14.6 ± 0.9 (13.3–16.0)
c’	3.2 (2.0–4.3)	-	2.6 (2.2–3.2)	-	3.4 ± 0.3 (2.9–3.8)	3.0 ± 0.3 (2.6–3.5)	3.0 ± 0.1 (2.9–3.1)	2.5 ± 0.2 (2.3–2.9)
V or T	62 (59–64)	-	62 (56–65)	-	63 ± 1.3 (62–65)	-	61.9 ± 3.2 (57.0–65.0)	39.2 ± 10.0 (25.0–54.0)
o	139 (121–167)	-	131 (108–171)	-	145 ± 12.8 (122–163)	145 (130–160)	119.3 ± 11.9 (106.7–133.3)	120.7 ± 5.0 (114.3–129.0)
DGO	-	-	-	-	-	-	17.6 ± 1.5 (16.0–20.0)	16.8 ± 0.6 (16.0–18.0)
Stylet length	13.6 (12.0–14.0)	12.5 (11.0–14.0)	14.4 (13.0–15.0)	13.0, 14.0	15.5 ± (15.0–16.5)	13.0 ± 0.8 (11.5–13.5)	14.8 ± 1.1 (13.0–16.0)	13.7 ± 0.6 (12.0–14.0)
Lip region width	-	-	-	-	-	-	6.7 ± 0.6 (6.0–7.0)	6.4 ± 0.4 (6.0–7.0)
Tail length	-	-	-	-	-	-	30.7 ± 1.2 (30.0–33.0)	30.5 ± 1.5 (28.5–33.0)
h	4.9 (3.0–7.0)	7.0 (4.0–9.0)	6.5 (5.0–8.0)	7.0, 10.0	10.0 ± 2.3 (7.0–14.5)	10.5 ± 1.0 (9.5–11.5)	10.0 ± 0.5 (9.5–10.5)	9.8 ± 0.8 (9.0–10.5)
Spicule length	-	21.0 (18.0–25.0)	-	19.0, 21.0	-	19.0 ± 0.7 (18.5–20.0)	-	20.4 ± 0.5 (20.0–21.0)
Gubernaculum length	-	7.0 (4.0–9.0)	-	8.0, 9.0	-	7.0 ± 0.4 (6.5–7.5)	-	7.4 ± 0.5 (7.0–8.0)

^a^ a = body length/maximum body width; b = body length/pharyngeal length; c = body length/tail length; c’ = tail length/body width at anus; DGO = distance from stylet base to dorsal gland opening; h = hyaline tail region length; o = (DGO/stylet length) × 100; V = (distance from anterior end to vulva/body length) × 100.

**Table 5:** Measurements of immature females and males of *Rotylenchulus borealis (= R. macrosoma)* from type locality in Israel and several localities in Spain. All measurements are in micrometers and in the form mean ± SD (range).

	Olive, Hulda, Israel, Type Population [10]		Paratypes from WANECO and USDA NEMATODE Collections, Re-Measured in This Study		Olive, Growth Chamber Built Population [18]	Wild Olive, Vejer, Cádiz, Spain [13]		Cultivated Olive, Jerez de la Frontera, Cádiz, Spain [3]	
Character/Ratio	Immature Females	Males	Immature females	Males	Immature Females	Immature Females	Males	Immature Females	Male
n	21	21	2	2	11	12	11	6	3
L ^a^	520–640	500–568	612, 634	493, 517	490 (470–510)	453 ± 28 (408–510)	467 ± 13 (449–495)	476 ± 26 (438–502)	475.0 ± 28 (446–501)
a	30–38	30–41	27.6, 37.1	32.9, 39.5	26.8 (24.5–29.5)	29.8 ± 2.1 (26.3–34.2)	31.5 ± 2.1 (27.5–34.0)	30.1 ± 1.2 (28.7–31.4)	31.7 ± 0.3 (31.3–31.9)
b	3.8–5.7	3.7–5.7	3.6, 4.1	3.8, 5.2	3.5 (3.0–3.8)	3.9 ± 0.3 (3.5–4.4)	4.7 ± 0.7 (3.5–5.2)	3.7 ± 0.3 (3.3–4.3)	3.4 ± 0.3 (3.1–3.6)
c	12–16	12–16	14.6, 15.3	12.0, 12.6	12.7 (11.8–14.7)	13.5 ± 1.3 (11.7–16.8)	13.9 ± 0.6 (13.1–15.0)	15.6 ± 1.3 (13.8–17.6)	15.2 ± 0.3 (14.9–15.4)
c’	3.7–5.0	-	3.5, 4.2	3.5, 3.9	-	3.7 ± 0.5 (2.8–4.4)	3.2 ± 0.4 (2.6–3.9)	3.3 ± 0.4 (2.8–4.0)	3.1 ± 0.2 (3.0–3.3)
V or T	63.0–68.0	20–33	62.3, 65.5	21.9, 28.6	62.1 (58.9–63.3)	62 ± 2 (59–64)	33 ± 5 (25–42)	61.5 ± 1.9 (59.0–64.0)	25.0 ± 4.1 (20.9–29.1)
o	139.0–188.0	-	126.5, 142.9	138.5, 141.0	134.4 (122.0–140.1)	152 ± 15 (126–183)	171 ± 15 (142–188)	135.8 ± 17.4 (116.0–156.0)	147.1 ± 8.5 (137.5–154.0)
DGO	-	-	21.5, 25.0	18.0, 19.0	-	25 ± 2 (22–27)	23 ± 2 (19.0–26.0)	24.0 ± 1.4 (22.0–26.0)	21.0 ± 1.0 (20.0–22.0)
Stylet length	18.0–22.0	13.0–16.0	17.0, 17.5	13.0, 13.5	19.7 (18.2–21.1)	16 ± 1 (15–18)	14 ± 1 (12–15)	17.8 ± 1.7 (16.0–20.0)	15.0 ± 1.0 (14.0–16.0)
Lip region width	-	-	-	5.0, 6.5	-	-	-	6.6 ± 0.7 (6.0–7.0)	6.3 ± 0.6 (6.0–7.0)
Tail length	-	-	41.5, 42.0	39.0, 43.0	-	34 ± 4 (26–40)	34 ± 2 (30–36)	30.8 ± 3.5 (27.0–36.0)	31.3 ± 1.5 (30.0–33.0)
h	13.0–18.0	15.0–23.0	18.5	10.0, 12.0	14.6 11.5–18.2)	10 ± 1 (9–12)	11 ± 1 (10–12)	10.5 ± 1.4 (9.0–12.0)	10.0 ± 1.0 (9.0–11.0)
Spicule length	-	21.0–24.0	-	21.0, 21.5		-	22 ± 2 (19–25)	-	21.3 ± 1.5 (20.0–23.0)
Gubernaculum length	-	8.0–10.0	-	6.5, 7.0		-	9 ± 1 (8–10)	-	9.0 ± 1.0 (8.0–10.0)

^a^ a = body length/maximum body width; b = body length/pharyngeal length; c = body length/tail length; c’ = tail length/body width at anus; DGO = distance from stylet base to dorsal gland opening; h = hyaline tail region length; o = (DGO/stylet length) × 100; V = (distance from anterior end to vulva/body length) × 100.

**Table 6:** Measurements of immature females and males of *Rotylenchulus borealis (= R. macrosoma)* from cultivated olive in Spain and Crete, Greece. All measurements are in micrometers and in the form mean ± SD (range).

	Cultivated Olive, Huévar del Aljarafe, Seville Province, Spain [3]		Cultivated Olive, Petrokefali, Crete, Greece [9]		Cultivated Olive, Limnes, Crete, Greece [9]	
Character/Ratio	Immature Females	Males	Immature Females	Males	Immature Females	Male
n	10	10	10	5	10	10
L ^a^	484 ± 30 (432–520)	478 ± 31 (432–514)	467 ± 27 (432–506)	468 ± 31 (433–503)	488 ± 31 (428–526)	463 ± 33 (418–516)
a	29.7 ± 1.5 (27.6–32.1)	29.9 ± 1.4 (27.6–31.9)	28.9 ± 1.9 (26.1–31.6)	30.8 ± 2.1 (27.1–32.0)	29.7 ± 1.0 (28.5–31.4)	28.2 ± 1.8 (26.1–31.3)
b	3.7 ± 0.3 (3.3–4.3)	3.6 ± 0.3 (3.0–4.1)	3.6 ± 0.3 (3.3–4.3)	3.4 ± 0.3 (3.1–3.6)	3.7 ± 0.2 (3.4–4.1)	3.6 ± 0.3 (3.2–4.0)
c	15.4 ± 1.2 (14.1–17.6)	14.8 ± 0.7 (13.4–15.4)	15.3 ± 0.9 (13.8–17.0)	14.7 ± 0.9 (13.1–15.4)	15.4 ± 1.0 (14.1–17.1)	13.8 ± 1.3 (11.6–15.4)
c’	3.1 ± 0.3 (2.6–4.0)	3.0 ± 0.1 (2.8–3.1)	3.3 ± 0.3 (2.8–4.0)	3.0 ± 0.2 (2.8–3.3)	3.1 ± 0.4 (2.6–3.8)	2.9 ± 0.1 (2.8–3.1)
V or T	62.6 ± 2.2 (59.0–66.0)	27.1 ± 3.8 (21.1–32.2)	61.4 ± 2.0 (58.0–64.0)	31.8 ± 1.3 (30.0–33.0)	62.4 ± 1.9 (60.0–65.0)	31.3 ± 5.3 (21.5–37.1)
o	138.0 ± 10.4 (126.3–156.3)	135.1 ± 3.8 (129.4–140.0)	129.8 ± 16.6 (105.0–156.3)	142.5 ± 7.2 (133.3–153.0)	128.8 ± 8.4 (116.7–138.0)	140.3 ± 5.2 (133.3–147.0)
DGO	23.8 ± 1.3 (22.0–26.0)	21.6 ± 1.1 (20.0–23.0)	23.8 ± 1.5 (21.0–26.0)	20.8 ± 1.3 (20.0–23.0)	23.6 ± 1.3 (21.0–25.0)	21.6 ± 1.7 (19.0–24.0)
Stylet length	17.3 ± 1.2 (16.0–19.0)	16.0 ± 0.9 (15.0–17.0)	18.5 ± 1.7 (16.0–21.0)	14.6 ± 0.5 (14.0–15.0)	17.4 ± 1.4 (15.5–20.0)	15.4 ± 0.5 (15.0–16.0)
Lip region width	6.6 ± 0.6 (6.0–7.5)	6.5 ± 0.5 (6.0–7.0)	6.7 ± 0.7 (6.0–7.5)	6.6 ± 0.5 (6.0–7.0)	6.7 ± 0.8 (6.0–8.0)	6.5 ± 0.6 (6.0–7.5)
Tail length	31.5 ± 3.2 (27.0–37.0)	32.4 ± 2.3 (29.0–35.0)	30.6 ± 2.8 (27.0–36.0)	31.4 ± 0.9 (30.0–32.0)	31.8 ± 3.0 (28.0–37.0)	33.6 ± 2.4 (29.0–37.0)
h	10.8 ± 1.6 (9.0–13.0)	10.6 ± 0.5 (10.0–11.0)	10.4 ± 1.1 (9.0–12.0)	10.2 ± 1.3 (9.0–11.0)	10.6 ± 1.3 (9.0–12.0)	10.5 ± 0.8 (9.0–12.0)
Spicule length	-	22.6 ± 1.4 (21.0–24.0)	-	21.8 ± 1.3 (20.0–23.0)	-	22.4 ± 1.4 (20.0–24.0)
Gubernaculum length	-	10.0 ± 0.9 (9.0–11.0)	-	9.2 ± 0.8 (8.0–10.0)	-	10.2 ± 0.8 (9.0–11.0)

^a^ a = body length/maximum body width; b = body length/pharyngeal length; c = body length/tail length; c’ = tail length/body width at anus; DGO = distance from stylet base to dorsal gland opening; h = hyaline tail region length; o = (DGO/stylet length) × 100; V = (distance from anterior end to vulva/body length) × 100.

**Table 7:** Measurements of immature females and males of *Rotylenchulus borealis (= R. macrosoma)* from potato in Rwanda and almond-peach rootstock and corn from several localities in Europe. All measurements are in micrometers and in the form mean ± SD (range).

	Potato, Burera, North Rwanda [14]		Almond-Peach Rootstock, Montañana, Zaragoza, Spain, This Study		Corn, Bečej, Vojvodina, Serbia, This Study		Corn, Moretta, Cuneo, Italy, This Study	
Character/Ratio	Immature Females	Males	Immature Females	Males	Immature Females	Males	Immature Females	Males
n	6	1	10	10	10	10	10	10
L ^a^	403 ± 8 (395–416)	462	461 ± 36 (401–517)	482 ± 33 (410–533)	425 ± 13 (401–440)	461 ± 39 (400–505)	427 ± 11 (411–441)	436.7 ± 28 (405–483)
a	24.3 ± 0.7 (23.5–25.5)	29.2	27.6 ± 2.4 (25.1–31.9)	28.1 ± 1.2 (26.5–30.4)	26.2 ± 1.4 (24.3–28.2)	25.5 ± 1.6 (22.1–27.3)	25.9 ± 1.7 (23.0–28.0)	27.6 ± 1.8 (25.3–31.2)
b	4.1 ± 0.1 (4.0–4.3)	4.1	3.1 ± 0.3 (2.6–3.6)	4.1 ± 0.2 (3.8–4.6)	3.5 ± 0.4 (2.9–4.2)	3.9 ± 0.4 (3.4–4.6)	3.6 ± 0.4 (2.9–4.2)	3.9 ± 0.2 (3.0–3.3)
c	13.1 ± 0.7 (12.4–14.2)	13.6	12.6 ± 1.3 (11.0–14.9)	13.7 ± 1.7 (11.0–17.6)	12.6 ± 0.7 (12.0–14.0)	15.3 ± 1.4 (13.7–17.9)	12.6 ± 0.8 (11.8–14.5)	13.2 ± 0.9 (12.3–14.6)
c’	2.9 ± 0.3 (2.5–3.3)	2.5	3.5 ± 0.3 (2.8–4.0)	2.9 ± 0.3 (2.4–3.6)	3.7 ± 0.2 (3.4–3.9)	2.7 ± 0.2 (2.4–3.0)	3.6 ± 0.2 (3.2–3.8)	3.1 ± 0.1 (3.0–3.3)
V or T	63.3 ± 1.2 (62.1–64.9)	-	61.1 ± 0.9 (59.6–62.7)	27.1 ± 5.3 (18.7–35.1)	59.9 ± 0.6 (59–61)	30.5 ± 3 (26–35)	60.1 ± 1.0 (58.0–61.8)	23.4 ± 2.1 (21.5–26.0)
o	-	-	127.4 ± 9.6 (112.5–141.2)	137.4 ± 15.4 (117.9–163.0)	120 ± 7 (113.3–135.5)	131 ± 10 (102–133)	118.0 ± 7.9 (106.3–129.0)	122.7 ± 7.9 (114.3–143.0)
DGO	20.2 ± 1.6 (18.0–22.0)	-	20.8 ± 2.4 (17.5–24.0)	19.1 ± 2.1 (16.0–22.0)	17.9 ± 1.6 (16.0–21.0)	18.6 ± 1.1 (17.0–21.0)	17.7 ± 1.5 (16.0–20.0)	17.4 ± 1.0 (16.0–20.0)
Stylet length	17.3 ± 0.3 (17.0–18.0)	16.8	16.3 ± 0.8 (15.0–17.0)	13.9 ± 0.3 (13.5–14.5)	14.9 ± 0.6 (14.0–16.0)	14.2 ± 0.3 (13.5–14.5)	15.0 ± 0.7 (14.0–16.0)	14.2 ± 0.4 (13.5–15.0)
Lip region width	-	-	6.6 ± 0.4 (6.0–7.0)	6.1 ± 0.4 (5.5–6.50)	6.5 ± 0.3 (6.0–7.0)	-	6.5 ± 0.3 (6.0–7.0)	6.5 ± 0.4 (6.0–7.0)
Tail length	30.9 ± 1.3 (29.0–32.0)	34.0	37.1 ± 5.1 (27.0–41.5)	35.6 ± 5.0 (25.5–43.0)	33.8 ± 1.5 (31.0–35.5)	30.3 ± 4.1 (23.5–35.0)	33.9 ± 2.0 (30.0–36.5)	33.6 ± 1.3 (32.0–36.0)
h	-	-	12.3 ± 1.6 (9.5–14.0)	11.6 ± 2.0 (7.5–14.5)	12.2 ± 1.4 (10.5–15.0)	8.7 ± 1.2 (7.0–10.0)	12.5 ± 1.7 (10.5–15.0)	8.5 ± 0.9 (7.0–10.0)
Spicule length	-	-	-	21.9 ± 1.2 (20.0–23.5)	-	22.3 ± 1.3 (20.0–24.5)	-	22.9 ± 0.7 (22.0–24.0)
Gubernaculum length	-	-	-	9.2 ± 1.0 (8.0–11.0)	-	8.0 ± 0.7 (7.0–9.0)	-	7.7 ± 0.3 (7.0–8.0)

^a^ a = body length/maximum body width; b = body length/pharyngeal length; c = body length/tail length; c’ = tail length/body width at anus; DGO = distance from stylet base to dorsal gland opening; h = hyaline tail region length; o = (DGO/stylet length) × 100; V = (distance from anterior end to vulva/body length) × 100.

**Table 8:** Measurements of immature females and males of *Rotylenchulus borealis (= R. macrosoma)* from corn and wheat from several localities in Europe. All measurements are in micrometers and in the form mean ± SD (range).

	Corn, Le Sen, Landes, France, This Study		Wheat, Mihail Kogalniceau, Romania, This Study		Corn, Létavertes, Hajdú-Bihar, Hungary, This Study		Corn, Möckmühl, Heilbronn, Germany, This Study	
Character/Ratio	Immature Females	Males	Immature Females	Males	Immature Females	Males	Immature Females	Males
n	10	10	10	10	10	10	10	10
L ^a^	428 ± 22 (396–473)	444 ± 21 (411–477)	435 ± 29 (387–468)	435 ± 25 (409–485)	430 ± 14 (412–458)	432 ± 19.3 (411–474)	422 ± 16 (409–429)	417 ± 7 (409–429)
a	26.3 ± 1.3 (24.2–28.3)	28.3 ± 1.8 (25.7–31.2)	27.3 ± 1.4 (24.9–29.1)	28.1 ± 1.8 (25.6–30.3)	27.0 ± 0.8 (25.8–28.3)	28.9 ± 2.5 (26.3–32.2)	27.3 ± 0.7 (26.2–28.3)	27.6 ± 1.6 (25.6–29.8)
b	3.6 ± 0.4 (3.2–4.3)	3.9 ± 0.2 (3.6–4.1)	3.7 ± 0.3 (3.3–4.2)	3.8 ± 0.2 (3.5–4.0)	3.7 ± 0.1 (3.6–4.0)	3.8 ± 0.1 (3.5–4.0)	3.6 ± 0.1 (3.4–3.8)	3.7 ± 0.2 (3.4–4.1)
c	12.8 ± 0.8 (11.4–14.3)	13.5 ± 0.5 (12.6–14.0)	14.0 ± 0.9 (12.6–15.5)	13.0 ± 0.5 (12.6–14.0)	14.1 ± 0.4 (13.5–14.8)	14.1 ± 1.0 (12.4–15.4)	14.0 ± 0.2 (13.7–14.3)	13.1 ± 1.0 (11.6–15.3)
c’	3.2 ± 0.2 (2.9–3.4)	3.0 ± 0.2 (2.7–3.2)	3.0 ± 0.2 (2.8–3.3)	3.1 ± 0.2 (2.8–3.3)	3.0 ± 0.1 (2.9–3.3)	2.9 ± 0.2 (2.7–3.3)	3.0 ± 0.1 (2.9–3.3)	3.1 ± 0.2 (2.8–3.3)
V or T	60.5 ± 0.8 (59.5–62.0)	31.0 ± 6.8 (26.5–43.0)	60.3 ± 0.9 (58.1–61.5)	32.2 ± 3.1 (29.5–37.1)	60.6 ± 0.5 (60.0–61.5)	33.9 ± 7.4 (27.0–48.1)	60.6 ± 0.5 (60.0–61.5)	33.9 ± 7.4 (27.0–48.1)
o	120.1 ± 7.3 (106.7–130.0)	117.6 ± 6.9 (107.1–129.0)	124.0 ± 9.7 (106.7–135.5)	124.5 ± 8.5 (113.3–138.0)	121.3 ± 8.8 (106.7–137.9)	125.5 ± 7.6 (115.4–138.0)	125.0 ± 7.5 (113.3–135.7)	121.5 ± 6.6 (107.1–129.0)
DGO	17.9 ± 1.5 (16.0–20.0)	16.8 ± 0.91 (15.0–18.0)	19.0 ± 1.9 (16.0–21.0)	17.5 ± 0.6 (17.0–18.5)	18.3 ± 1.3 (16.0–20.0)	17.3 ± 1.0 (15.0–18.5)	18.6 ± 1.1 (17.0–20.0)	17.0 ± 0.8 (15.0–18.0)
Stylet length	14.9 ± 0.6 (14.0–16.0)	14.3 ± 0.9 (14.0–15.0)	15.3 ± 0.5 (14.5–16.0)	14.1 ± 0.6 (13.0–15.0)	15.1 ± 0.4 (14.5–15.5)	13.8 ± 0.5 (13.0–14.5)	14.9 ± 0.6 (14.0–15.5)	14.0 ± 0.4 (13.5–15.0)
Lip region width	6.6 ± 0.3 (6.0–7.0)	6.5 ± 0.3 (6.0–7.0)	6.6 ± 0.2 (6.5–7.0)	6.5 ± 0.4 (6.0–7.0)	6.7 ± 0.3 (6.5–7.0)	6.6 ± 0.4 (6.0–7.0)	6.6 ± 0.3 (6.0–7.0)	6.4 ± 0.5 (6.0–7.0)
Tail length	33.5 ± 1.4 (31.0–35.5)	33.3 ± 0.7 (32.0–34.0)	31.0 ± 1.5 (29.0–34.0)	31.8 ± 1.9 (32.0–38.0)	30.6 ± 1.3 (29.0–33.0)	31.2 ± 2.3 (28.0–36.0)	30.1 ± 1.0 (28.0–31.0)	32.5 ± 2.0 (28.0–36.0)
h	11.4 ± 0.8 (10.5–12.5)	10.0 ± 1.5 (8.0–15.5)	10.4 ± 0.6 (9.5–11.5)	10.3 ± 1.8 (8.0–12.5)	10.1 ± 0.4 (9.5–10.5)	9.9 ± 0.6 (9.0–11.0)	10.0 ± 0.4 (9.5–10.5)	9.7 ± 1.3 (8.0–12.5)
Spicule length	-	22.5 ± 0.6 (22.0–23.5)	-	22.2 ± 0.3 (22.0–23.0)	-	21.8 ± 2.5 (21.0–22.5)	-	21.6 ± 0.8 (20.0–22.5)
Gubernaculum length	-	7.8 ± 0.3 (7.5–8.0)	-	7.7 ± 0.4 (7.0–8.0)	-	7.5 ± 0.4 (7.0–8.0)	-	7.4 ± 0.4 (7.0–8.0)

^a^ a = body length/maximum body width; b = body length/pharyngeal length; c = body length/tail length; c’ = tail length/body width at anus; DGO = distance from stylet base to dorsal gland opening; h = hyaline tail region length; o = (DGO/stylet length) × 100; V = (distance from anterior end to vulva/body length) × 100.

## Data Availability

The datasets generated during and/or analysed during the current study are available from the corresponding author on reasonable request.

## Supplementary Materials

The following material is available online at https://www.mdpi.com/2223-7747/10/1/7/s1. Figure S1. BIOCLIM variables for *Rotylenchulus* species with ≥ 3 reports. BIO3 [Isothermality, (BIO2/BIO7) * 100], BIO4 [Temperature seasonality, (SD * 100)], BIO7 [Temperature annual range (BIO5-BIO6)], BIO9 (mean temperature of driest quarter), BIO10 (mean temperature of the warmest quarter), BIO 15 [precipitation seasonality (CV)], BIO17 (precipitation of driest quarter), and BIO18 (precipitation of the warmest quarter).
