# Supplementary material for: Global Distribution of the Reniform Nematode Genus Rotylenchulus with the Synonymy of Rotylenchulus macrosoma with Rotylenchulus borealis
Source: Plants (Basel). 2020 Dec 23;10(1):7. doi: 10.3390/plants10010007 (PMC7822487; doi:10.3390/plants10010007)
Supplement: Supplementary file 1 [file plants-10-00007-s001.pdf]

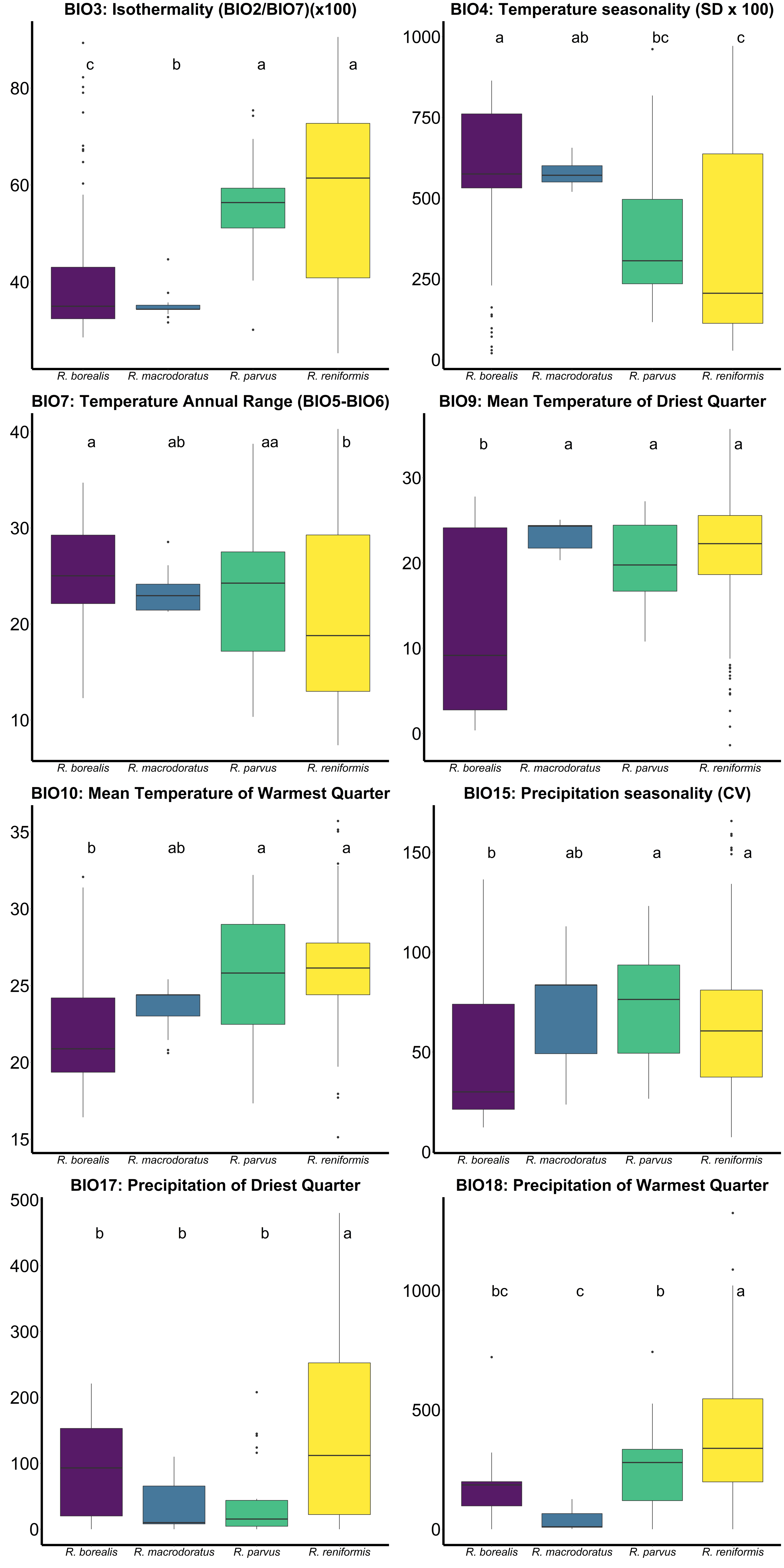
BIO3: Isothermality (BIO2/BIO7)(x100)
80
60
40
c
b
a
a
R. borealis
R. macrodoratus
R. parvus
R. reniformis
BIO4: Temperature seasonality (SD x 100)
1000
750
500
250
0
a
ab
bc
c
R. borealis
R. macrodoratus
R. parvus
R. reniformis
BIO7: Temperature Annual Range (BIO5-BIO6)
40
30
20
10
a
ab
aa
b
R. borealis
R. macrodoratus
R. parvus
R. reniformis
BIO9: Mean Temperature of Driest Quarter
30
20
10
0
b
a
a
a
R. borealis
R. macrodoratus
R. parvus
R. reniformis
BIO10: Mean Temperature of Warmest Quarter
35
30
25
20
15
b
ab
a
a
R. borealis
R. macrodoratus
R. parvus
R. reniformis
BIO15: Precipitation seasonality (CV)
150
100
50
0
b
ab
a
a
R. borealis
R. macrodoratus
R. parvus
R. reniformis
BIO17: Precipitation of Driest Quarter
500
400
300
200
100
0
b
b
b
a
R. borealis
R. macrodoratus
R. parvus
R. reniformis
BIO18: Precipitation of Warmest Quarter
1000
500
0
bc
c
b
a
R. borealis
R. macrodoratus
R. parvus
R. reniformis
